# Coordination-Crosslinked Carboxymethyl Chitosan-Mn^2+^ Hydrogel: Structure–Activity Relationship for In Vitro cGAS-STING Immune Stimulation

**DOI:** 10.3390/molecules31213891

**Published:** 2026-10-28

**Authors:** Yue Yang, Xiaoxin Chen, Shijie Li, Yuncai Xiao, Xianwen Hu

**Affiliations:** 1 College of Chemistry, Huazhong Agricultural University, Wuhan 430070, China; m15102650928@163.com (Y.Y.); 19117558396@163.com (X.C.); 2 College of Veterinary Medicine, Huazhong Agricultural University, Wuhan 430070, China; lishijie@webmail.hzau.edu.cn

**Keywords:** carboxymethyl chitosan, Manganese, cGAS-STING pathway, synergistic immunity, combination

## Abstract

Both carboxymethyl chitosan (CMCS) and Mn^2+^ can stimulate the cGAS-STING pathway to promote the production of immune factors such as type I interferons (I IFNs), thereby regulating the body’s immune response. In this study, a sustained-release Mn^2+^-loaded CMCS-Mn hydrogel was designed, and the synergistic effects of CMCS and Mn^2+^ on innate immunity, as well as factors influencing the immune response, were investigated. The optimal gel formulation was obtained with a CMCS concentration of 1 mg/mL and a MnCl_2_ concentration of 0.02 mol/L, yielding a gel with a particle size of 472.16 nm, a polydispersity index of 0.35, a Mn^2+^ content of 1.488 mg/g, and a specific surface area of 4.74 m^2^/g. The hydrogel forms mainly via coordination interactions between Mn^2+^ and the amino and carboxyl groups on CMCS molecular chains and exhibits pH-dependent Mn^2+^ release behavior. Cellular assays demonstrated that the combination of 600 μg/mL CMCS-Mn^2+^ hydrogel and 5 μg/mL free Mn^2+^ produced the strongest IFN-β induction. The hydrogel exhibits synergistic immunomodulatory effects of both CMCS and Mn^2+^; while the amino groups are critical for the immunological activity of CMCS, Mn^2+^ plays a more prominent role. Partial substitution of Mn^2+^ by Ca^2+^ can loosen the cross-linked network and further modulate Mn^2+^ dissociation from the hydrogel.

## 1. Introduction

The cGAS-STING pathway, an important component of the host’s innate immunity, plays a significant role in immunotherapy. It can be activated by double-stranded DNA (dsDNA) from bacteria, viruses, or tumor tissues to produce type I interferons (IFNs) [1,2,3]. Type I IFNs connect innate immunity with adaptive immunity. They not only act as broad-spectrum antiviral agents but also promote the maturation and migration of DCs, enhance the cytotoxicity mediated by cytotoxic T lymphocytes or natural killer cells, and protect effector cells from apoptosis [4,5,6]. Therefore, researchers have made considerable efforts to find suitable cGAS-STING agonists to develop vaccine adjuvants and anticancer drugs since the discovery of STING [7].

Manganese, one of the most abundant metals in mammalian tissues, plays an important role in a variety of physiological processes [8]. As a recently identified cGAS-STING agonist, Mn^2+^ can help the host resist infection by various DNA viruses and inhibit tumor cell growth [9,10]. However, manganese can also be toxic. Excessive manganese exposure can damage the central nervous system, trigger pathological immune activation and cause irreversible cell and tissue damage [11,12,13,14]. In addition, manganese is pH-sensitive and can exist in multiple valence states. To enhance the innate immunostimulatory activity of Mn^2+^ in the body, reduce its side effects and prevent its oxidation, a new carrier is needed to stabilize Mn^2+^ and control its release in the body.

CMCS is a chitosan derivative with good biocompatibility and biodegradability, hemostatic and antibacterial properties, the ability to promote cell proliferation, and immunoregulatory activity [15,16,17,18]. Its degradation products, such as glucosamine, can be absorbed or metabolized by the body without toxic side effects [19,20]. Its molecular chains contain abundant amino and carboxyl groups, providing numerous binding sites for metal ions, and CMCS is widely used in hydrogel preparation [15,21,22]. CMCS can stimulate the cGAS-STING pathway and enhance the body’s immune response [23,24].

In this study, CMCS-Mn hydrogel was prepared, and the gel formation mechanism was investigated. The ability of the gel to induce IFN-β was evaluated by measuring cellular IFN-β expression, and the synergistic activation of the cGAS-STING pathway by CMCS and Mn^2+,^ along with factors affecting IFN-β induction, was investigated.

## 2. Results and Discussion

### 2.1. Preparation and Characterization of CMCS-Mn Hydrogel

**Figure 1:**
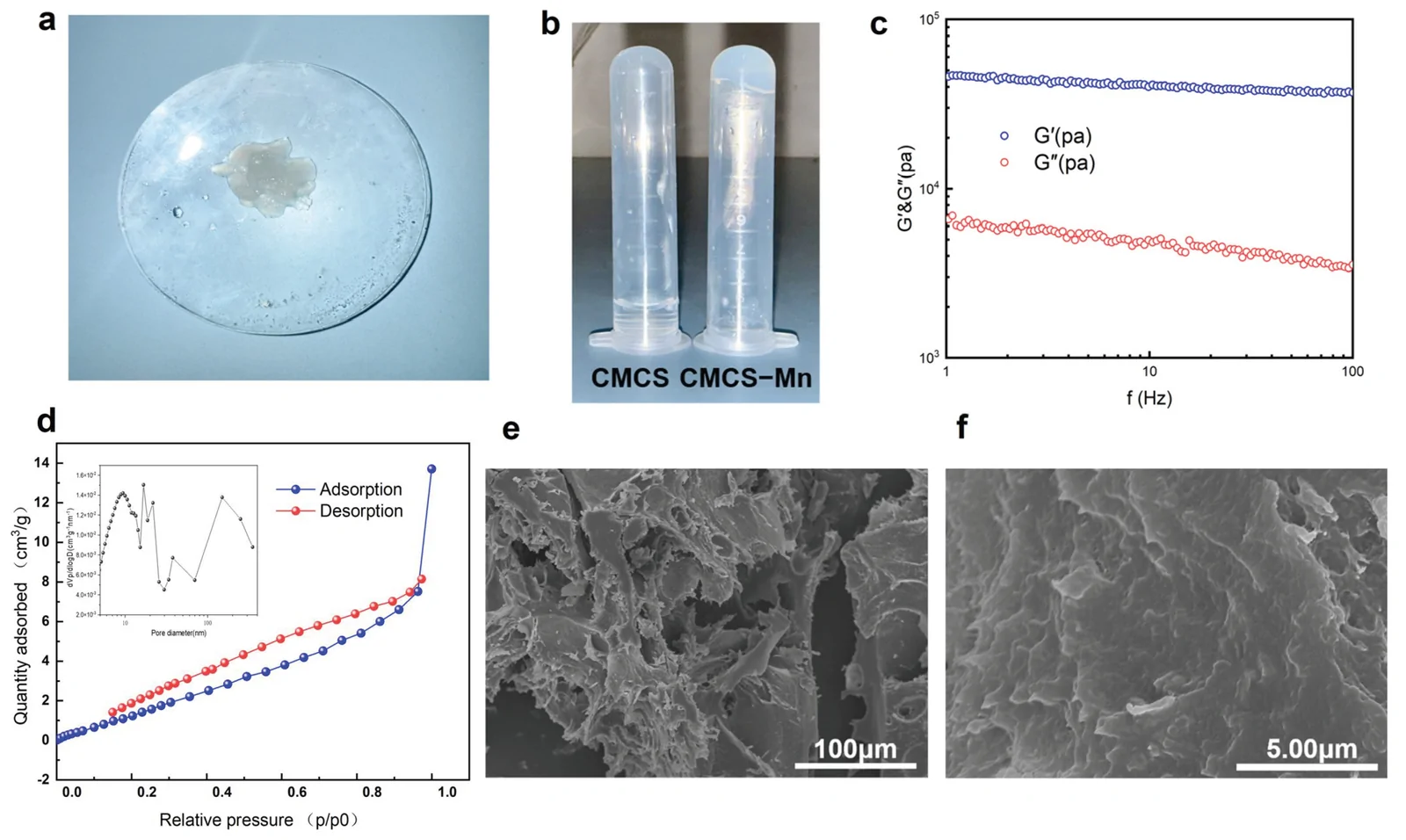
Characterization of the gel. (**a**) Morphology of the gel. (**b**) Inverted state of CMCS and CMCS-Mn samples. (**c**) Corresponding frequency sweep rheological curves. (**d**) N_2_ adsorption–desorption isotherm. Inset: NLDFT pore-size distribution. (**e**,**f**) SEM images of the gel.

A translucent hydrogel mass rapidly formed upon adding MnCl_2_ solution dropwise to CMCS solution; the resulting hydrogel had a soft texture and the typical semisolid consistency of hydrogels (Figure 1a). A digital inverted test verified the self-supporting property of CMCS-Mn hydrogel (Figure 1b). Frequency sweep rheology demonstrated that storage modulus (G′) was consistently higher than loss modulus (G″) over the whole tested frequency range, confirming the elastic hydrogel nature (Figure 1c).

The dry weight and Mn^2+^ content of hydrogels prepared with different concentrations of CMCS and MnCl_2_ are shown in Table 1. The mass fraction of Mn^2+^ in dried hydrogels varied slightly across different CMCS and MnCl_2_ concentrations, remaining at approximately 1%. Hydrogel formation results from the spontaneous binding between Mn^2+^ and CMCS. With sufficient reaction time, Mn^2+^ dominates the crosslinking process, resulting in a relatively constant Mn^2+^ content in dried hydrogels that is insensitive to precursor concentrations. To reduce raw material consumption, 1.0 mg/mL CMCS and 0.02 mol/L MnCl_2_ were selected as the optimal formulation. The manganese loading was 1.488 mg/g.

Dynamic light scattering (DLS) measurements from three replicate tests showed that the average hydrodynamic diameter of hydrated CMCS-Mn^2+^ gel particles was 472.00 ± 4.00 nm, with an average polydispersity index (PDI) of 0.35 ± 0.04. After freeze-drying, the samples were characterized by scanning electron microscopy (SEM). The dried nanoparticle aggregates stacked together, forming the layered macroscopic skeleton observed in SEM micrographs (Figure 1c,d, File S1). The average pore size within the gel skeleton calculated from SEM images was 99.9 ± 8.1 nm.

Nitrogen adsorption–desorption was used to characterize the porous structure of the freeze-dried hydrogel (Figure 1b). Before testing, the freeze-dried hydrogel sample (0.0330 g) was degassed under vacuum at 120 °C for 6 h. The type IV isotherm with an H3 hysteresis loop indicated slit-shaped mesopores originating from nanoparticle stacking. BET fitting on the adsorption branch (p/p_0_ = 0.055–0.30) gave a specific surface area of 4.74 m^2^/g (R^2^ = 0.977). The total pore volume was 0.0197 cm^3^/g. The pore-size distribution shown in the inset spans 2–100 nm and is dominated by mesopores (2–50 nm), supporting controlled release of Mn^2+^.

### 2.2. Construction Mechanism of CMCS-Mn Hydrogel

**Figure 2:**
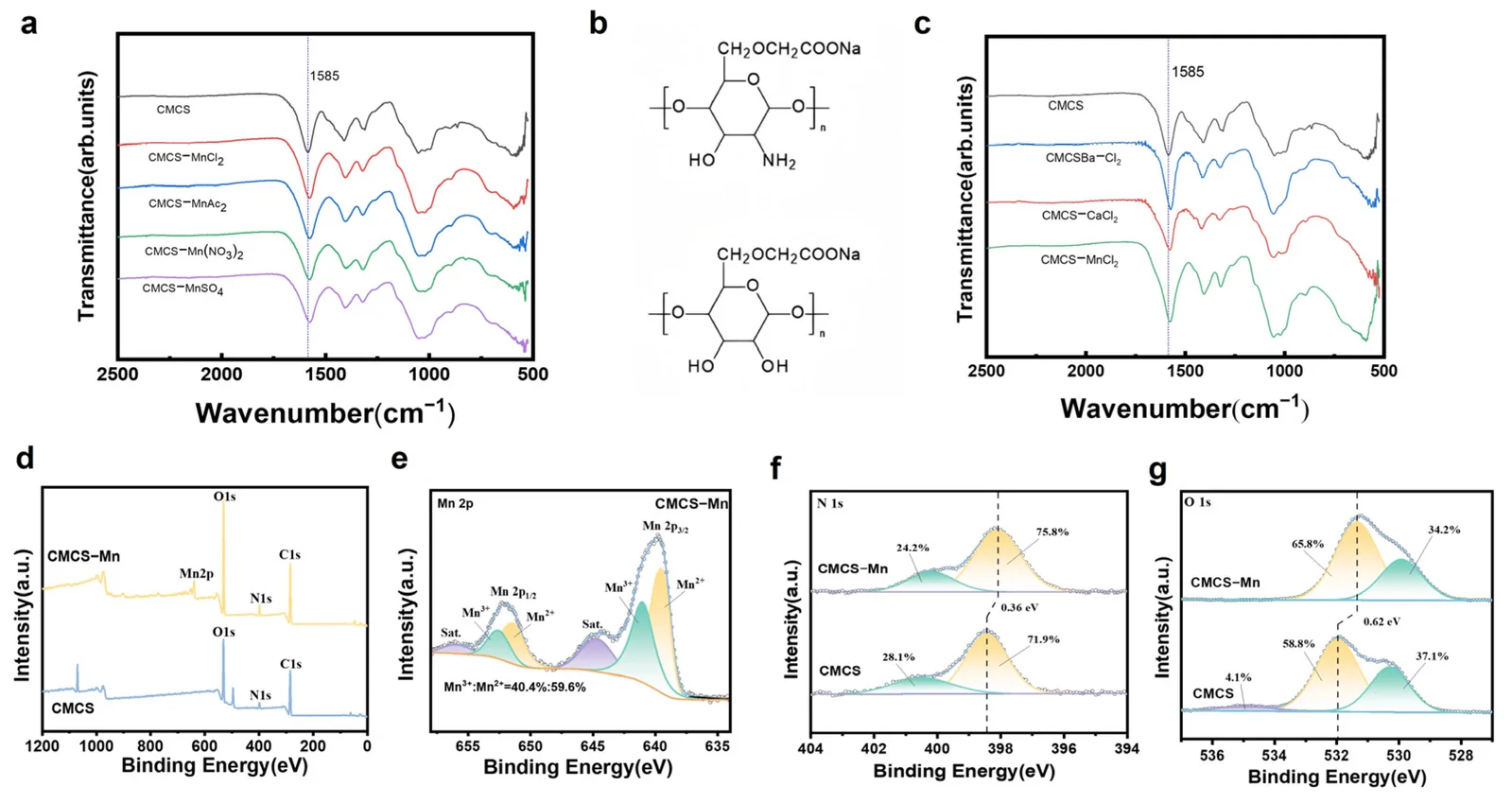
Structural characterization of CMCS and metal-ion-crosslinked CMCS materials. (**a**) FT-IR spectra of pristine CMCS and CMCS coordinated with various manganese salts. (**b**) Structural formulas of CMCS and CMC. (**c**) FT-IR spectra of CMCS coordinated with different divalent metal cations (Ba^2+^, Ca^2+^, Mn^2+^). (**d**) XPS survey spectra of CMCS and CMCS-Mn hydrogel. (**e**) Deconvoluted high-resolution Mn 2p XPS spectrum of CMCS-Mn. (**f**) High-resolution N 1s XPS spectra of CMCS and CMCS-Mn. (**g**) High-resolution O 1s XPS spectra of CMCS and CMCS-Mn.

To explore the anion dependence of CMCS-Mn gel formation, hydrogels were fabricated using manganese salts with various anions (Figure 2a). The asymmetric stretching peak of CMCS carboxylate groups at 1585 cm^−1^ shifts to ~1575–1576 cm^−1^ for all CMCS-Mn gels. The consistent peak shift demonstrates that anions have little effect on the binding between Mn^2+^ and CMCS. Moreover, all gels exhibit similar disintegration kinetics in EDTA-2Na solution and fully degrade within 6 h. Accordingly, gelation is mainly controlled by CMCS-Mn^2+^ interactions rather than salt anions.

The characteristic infrared band for asymmetric stretching of the carboxylate groups on CMCS molecular chains occurs at 1585 cm^−1^; it shifts to 1575 cm^−1^ in CMCS-CaCl_2_ gel, to 1577 cm^−1^ in CMCS-BaCl_2_ gel, and to 1575 cm^−1^ in CMCS-MnCl_2_ gel (Figure 2c). Upon coordination with metal ions, the C=O double-bond character of -COO^−^ weakens, while its C-O single-bond character increases. This leads to a more even distribution of electron density between the two C-O bonds and shifts the asymmetric carboxylate stretching band to a lower wavenumber. The infrared characterization results of the gels indicate alterations in the chemical environments of carboxyl and amino functional groups. Previous studies indicate that divalent metal ions rarely form continuous three-dimensional hydrogel networks through the carboxyl groups of CMC [25]. To help identify coordination sites in CMCS-Mn^2+^, CMC-Mn^2+^ was used as a control (Figure 2b). CMCS and CMC solutions of equal concentration and degree of substitution were separately added to MnCl_2_ solution of the same concentration. CMCS droplets formed gel spheres, while CMC droplets showed no obvious gelation. Nevertheless, potential differences in molecular weight, charge density and solubility between the two polymers may also influence their gelation behavior. Hence, this control experiment provides only supporting evidence and cannot independently verify the coordination between Mn^2+^ and amino groups.

The XPS survey spectrum (Figure 2d) confirms the successful incorporation of Mn into the CMCS matrix. The high-resolution Mn 2p spectrum (Figure 2e) reveals mixed-valence states of Mn^2+^ (59.6%) and Mn^3+^ (40.4%) in solid CMCS-Mn. The carboxyl oxygen and amino nitrogen of CMCS act as electron donors to coordinate with Mn centers. The ligand field effect splits the 3d orbitals of Mn ions, causing partial oxidation of Mn^2+^ to Mn^3+^. This mixed-valence state originates from coordination-induced electronic rearrangement.

Compared with CMCS, the main O 1s peak of CMCS-Mn (Figure 2g) shifts to higher binding energy by 0.62 eV, and the fraction of carboxyl oxygen increases from 58.8% to 65.8%. The N 1s peak (Figure 2f) shifts to higher binding energy by 0.36 eV, accompanied by a decrease in the proportion of amino nitrogen. During coordination, O and N atoms donate lone-pair electrons to Mn centers, reducing electron shielding and increasing binding energy. Simple physical adsorption cannot explain the simultaneous shifts in the O 1s and N 1s peaks and the changes in functional-group proportions.

Notably, Mn^3+^ detected by solid-state XPS only reflects the surface electronic state of the material. Mn^3+^ is unstable under physiological aqueous conditions, and no existing studies have confirmed its ability to activate the cGAS-STING pathway. Reported manganese-mediated innate immune activation has been attributed to Mn^2+^ [9,10,26]. Therefore, the immune response induced by CMCS-Mn mainly originates from Mn^2+^ released from the hydrogel.

These results suggest that Mn^2+^ in the gel mainly coordinates with the carboxyl and amino groups on the CMCS molecular chains, thereby acting as a crosslinking agent.

### 2.3. Concentration Screening

**Figure 3:**
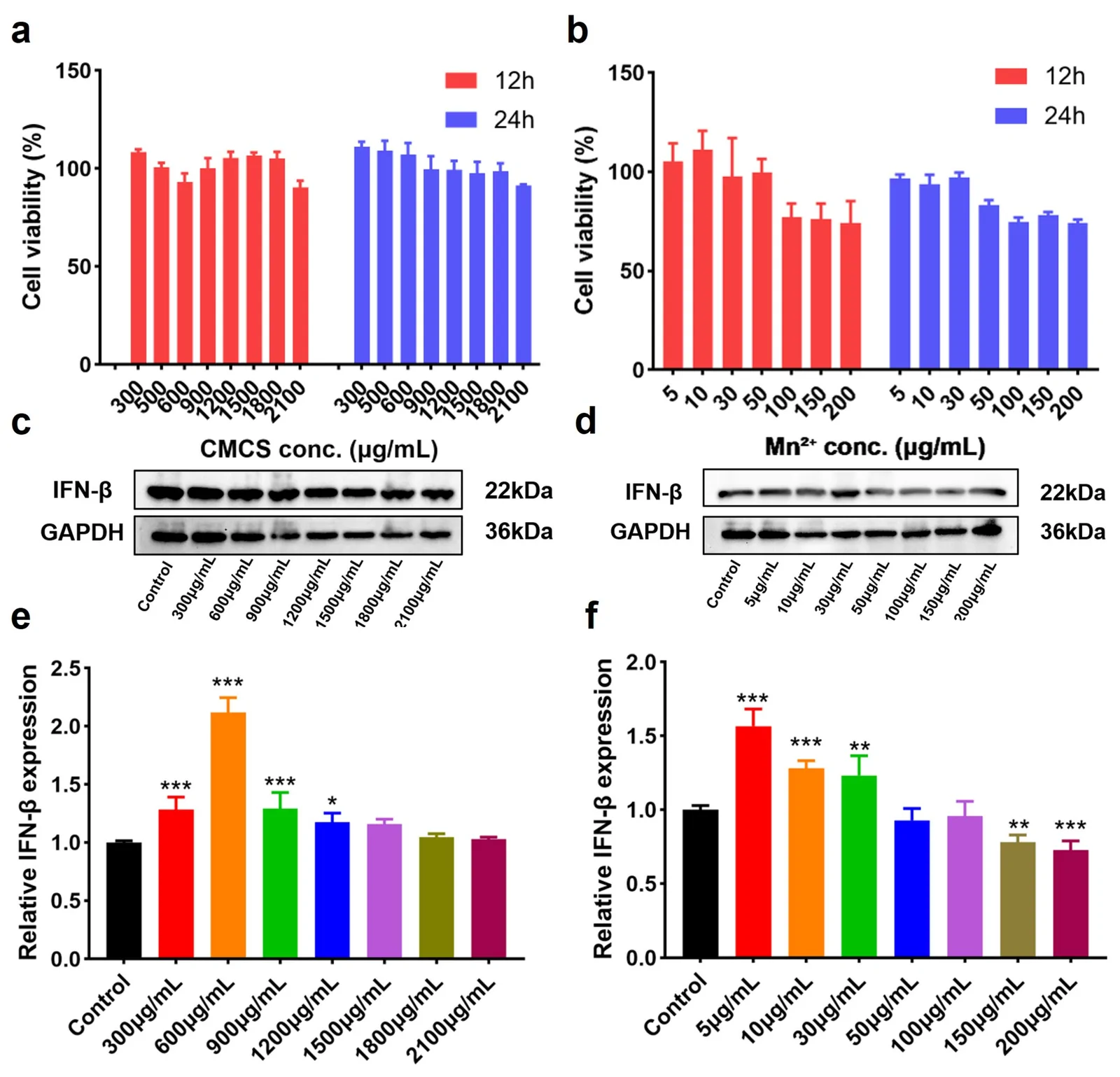
Cytotoxicity and IFN-β expression induced by CMCS and Mn^2+^ cell viability after 12 h and 24 h treatment with different concentrations of CMCS and Mn^2+^. (**a**) CMCS group; (**b**) Mn^2+^ group (*n* = 3). (**c**) Western blot of IFN-β for CMCS-Mn hydrogel groups. (**d**) Western blot of IFN-β for Mn^2+^ groups. (**e**,**f**) Quantitative analysis of relative IFN-β expression in (**c**,**d**) (*n* = 3). Data are presented as mean ± SD. *p*-values were calculated using ordinary one-way ANOVA with Dunnett’s multiple comparisons test. * *p* < 0.05, ** *p* < 0.01, *** *p* < 0.001.

The cytotoxicity of CMCS and Mn^2+^ was assessed by CCK-8 assay at 12 h and 24 h (Figure 3a,b). CMCS maintained cell viability > 90% from 300 to 2100 μg/mL, demonstrating good biocompatibility. By contrast, Mn^2+^ induced dose-dependent cytotoxicity: 5–30 μg/mL Mn^2+^ was non-toxic, and cell viability declined as concentration increased.

The effects of different concentrations of Mn^2+^ and CMCS-Mn hydrogel on IFN-β expression are shown (Figure 3c,d, File S2). Mn^2+^ exerted the strongest effect on IFN-β expression at a concentration of 5 μg/mL, approximately 1.6-fold that of the control group; the effect diminished as the concentration increased. CMCS-Mn hydrogel induced the highest IFN-β expression at a concentration of 600 μg/mL (approximately 2.1-fold that of the control group), with weaker effects at both lower and higher concentrations, indicating an initial increase followed by a decline as concentration increased. The optimal stimulation concentrations for Mn^2+^ and CMCS-Mn hydrogel were 5 μg/mL and 600 μg/mL, respectively.

### 2.4. Synergistic Immunostimulatory Effects of CMCS and Mn^2+^

To investigate whether Mn^2+^ and CMCS exhibit a synergistic immunostimulatory effect, four concentration combinations of CMCS and Mn^2+^ were selected near the optimal concentration range based on their respective effects on IFN-β expression: CMCS 300 μg/mL + Mn^2+^ 10 μg/mL, CMCS 300 μg/mL + Mn^2+^ 30 μg/mL, CMCS 900 μg/mL + Mn^2+^ 10 μg/mL, and CMCS 900 μg/mL + Mn^2+^ 30 μg/mL.

**Figure 4:**
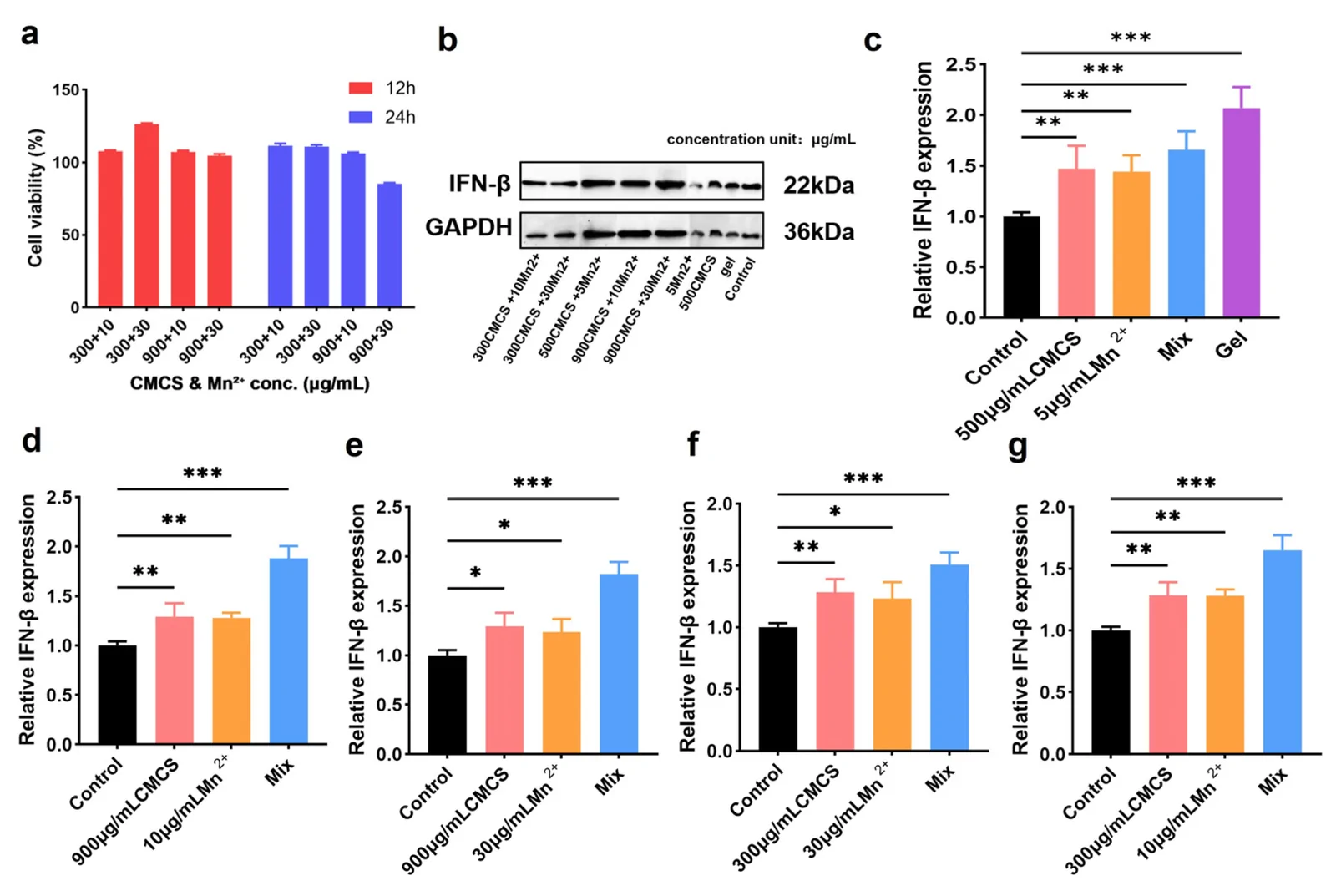
Cytotoxicity and IFN-β expression of CMCS combined with Mn^2+^. (**a**) Cell viability after co-treatment with CMCS and Mn^2+^ for 12 h and 24 h (*n* = 3). (**b**) Western blot bands of IFN-β and GAPDH. (**c**–**g**) Quantitative analysis of relative IFN-β expression under different treatment groups (*n* = 3). Data are presented as mean ± SD. *p*-values were calculated using ordinary one-way ANOVA with Dunnett’s multiple comparisons test. * *p* < 0.05, ** *p* < 0.01, *** *p* < 0.001.

The cytotoxicity of combined CMCS and Mn^2+^ was further verified. At the tested combined concentrations, cell viability remained high, confirming the biosafety of these combinations for subsequent protein expression assays (Figure 4a).

The IFN-β expression level in the Mn^2+^ 10 μg/mL experimental group was approximately 1.2-fold that of the control group; in the Mn^2+^ 30 μg/mL experimental group, it was approximately 1.2-fold; in the CMCS 300 μg/mL experimental group, it was approximately 1.3-fold; and in the CMCS 900 μg/mL experimental group, it was approximately 1.3-fold. At the selected concentrations of CMCS and Mn^2+^, the IFN-β expression levels in the experimental groups were moderately elevated, indicating modest immune activation. Specifically, the IFN-β expression level in the CMCS 300 μg/mL + Mn^2+^ 10 μg/mL experimental group was approximately 1.6-fold that of the control group; in the CMCS 300 μg/mL + Mn^2+^ 30 μg/mL group, it was approximately 1.5-fold; in the CMCS 900 μg/mL + Mn^2+^ 10 μg/mL group, it was approximately 1.9-fold; and in the CMCS 900 μg/mL + Mn^2+^ 30 μg/mL group, it was approximately 1.8-fold. All combinations of CMCS and Mn^2+^ increased IFN-β expression more than the respective individual solutions, suggesting a synergistic immunological effect between CMCS and Mn^2+^ (Figure 4d–f).

The hydrogel composition analysis in Section 2.1 yielded a CMCS-to-Mn^2+^ mass ratio of ~100:1. Based on this mass ratio from hydrogel digestion and the concentration-dependent effects of Mn^2+^ and CMCS on IFN-β expression in Section 2.3, 500 μg/mL CMCS and 5 μg/mL Mn^2+^ were chosen for subsequent investigation of the hydrogel’s immunostimulatory activity (Figure 4c). The IFN-β expression level in the 5 μg/mL Mn^2+^ experimental group was approximately 1.4-fold that of the control group; in the 500 μg/mL CMCS experimental group, it was approximately 1.5-fold; in the 500 μg/mL CMCS + 5 μg/mL Mn^2+^ experimental group, it was approximately 1.7-fold; and in the gel experimental group, it was approximately 2.1-fold that of the control group. Comparison of CMCS solution, MnCl_2_ solution, their mixture, and gel samples showed that the hydrogel induced higher IFN-β expression than the mixture, indicating strong IFN-β-inducing activity and the synergistic action of CMCS and Mn^2+^.

To evaluate the synergistic immunostimulatory effect between CMCS and Mn^2+^, the Bliss independence model [27] was adopted with reference to the data shown in Figure 4f:

*E_exp_ = E_A_ + E_B_ − E_A_E_B_*

where *E_A_* and *E_B_* are the relative IFN-β expression of Mn^2+^-only and CMCS-only groups, respectively; *E_exp_* represents the expected effect under independent action. The Bliss combination index is defined as *CI_Bliss_* = *E_obs_*/*E_exp_*. Different from cytotoxic inhibition assays, *CI_Bliss_* indicates synergistic enhancement for cytokine-promoting responses.

The calculated *CI_Bliss_* value of the physical mixture was ~2.13, while that of CMCS-Mn hydrogel was ~2.63, demonstrating stronger synergistic IFN-β activation after hydrogel crosslinking.

### 2.5. Relationship Between the Duration of CMCS-Mn Hydrogel Immunostimulation and Mn^2+^ Release

**Figure 5:**
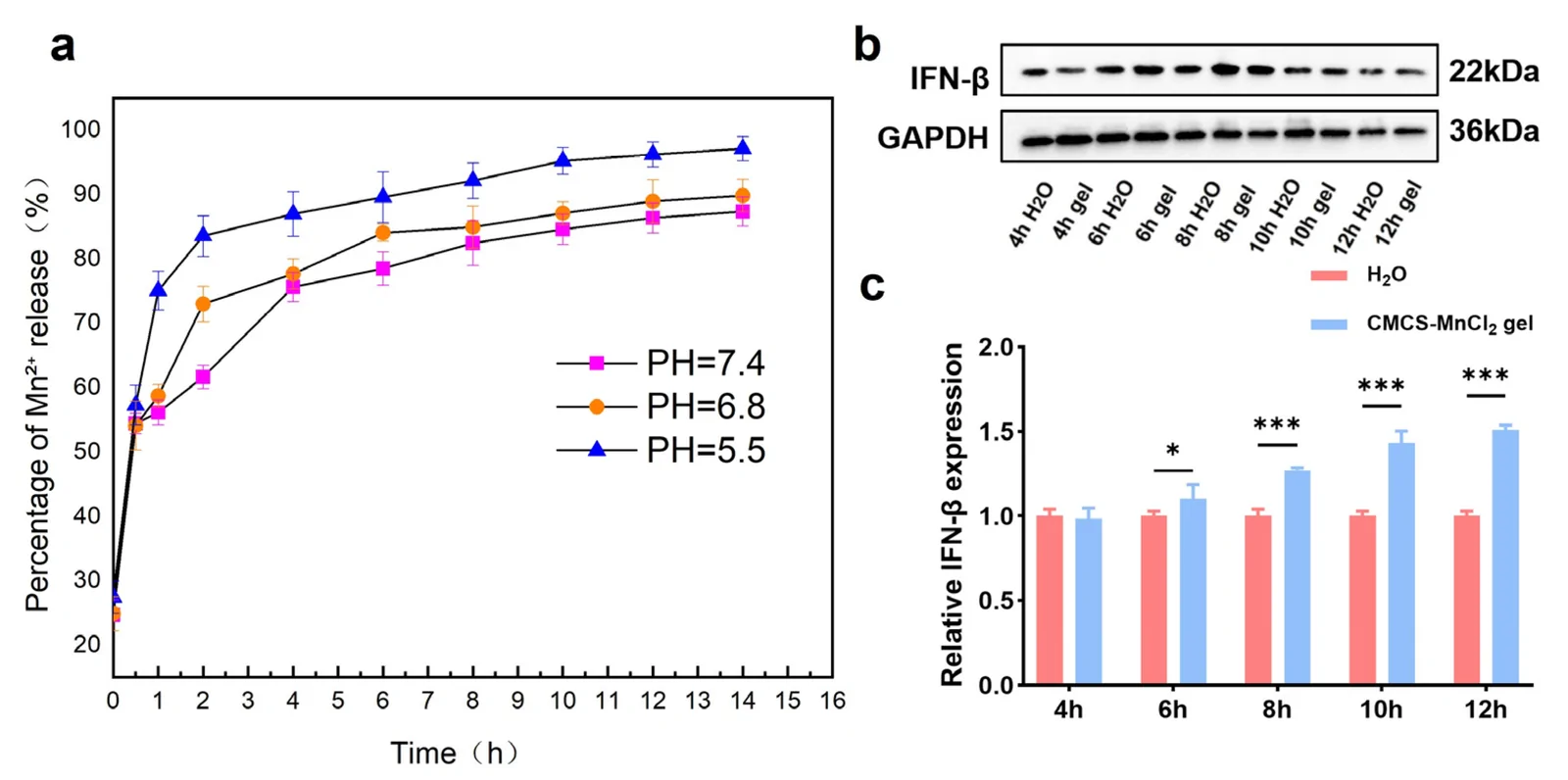
(**a**) Cumulative release profiles of Mn^2+^ from CMCS-Mn hydrogel at pH 5.5, 6.8 and 7.4. (**b**) Western blot bands of IFN-β under different stimulation durations. (**c**) Effects of different stimulation durations on IFN-β expression (*n* = 3). Data are presented as mean ± SD. *p*-values were calculated using ordinary one-way ANOVA with Dunnett’s multiple comparisons test. * *p* < 0.05, *** *p* < 0.001.

Under weakly acidic conditions, protonation of amino and carboxylate groups on CMCS competitively disrupts the coordination bonds with Mn^2+^ and accelerates early ion release. At all tested pH values, Mn^2+^ was rapidly released within the first 6 h, after which the release rate decreased and gradually approached a plateau, demonstrating pH-responsive release behavior (Figure 5a). At pH 7.4 (the physiological pH of normal tissues and interstitial fluid in vivo), the cumulative mass of Mn^2+^ released at equilibrium reached 1363.3 μg.

To further investigate the Mn^2+^ release behavior, the release data were fitted with the Korsmeyer–Peppas (KP) model. The Korsmeyer–Peppas model equation is expressed as:

$$\frac{M_{t}}{M_{\infty }}=kt^{n}$$

where $\frac{M_{t}}{M_{\infty }}$ represents the fraction of Mn^2+^ released at time *t*, *k* is the kinetic constant, *n* is the diffusion index used to determine the release mechanism.

Fitting results showed that the diffusion index n had values of 0.12172, 0.15624 and 0.15803 at pH 5.5, pH 6.8 and pH 7.4, respectively. All n values were less than 0.43, confirming that Mn^2+^ release was dominated by Fickian diffusion. Moreover, the rate constant k gradually decreased with increasing pH, indicating faster Mn^2+^ diffusion under acidic conditions. In acidic environments, carboxylate groups on CMCS become protonated and convert into carboxylic acid groups, weakening the coordination interaction between carboxylate groups and Mn^2+^. More Mn^2+^ dissociates from the hydrogel network into free ions, thereby accelerating Mn^2+^ release.

The material typically acts through this signaling pathway for more than 4 h. The Mn^2+^ release data showed that Mn^2+^ was released rapidly during the first 6 h, after which release gradually approached a plateau. The effect of stimulation duration on IFN-β expression largely followed the rate of Mn^2+^ release from the gel. No significant difference in IFN-β expression was observed at 4 h, while the stimulatory effect gradually intensified from 6 h to 12 h along with the release of Mn^2+^ (Figure 5b,c). The correlation between Mn^2+^ release kinetics and IFN-β expression indicates that, during gel-induced stimulation of the cGAS-STING pathway, Mn^2+^—as a key agonist—exerts a stronger effect than the carrier material CMCS.

### 2.6. Factors Affecting the Immunological Effect of CMCS-Mn

**Figure 6:**
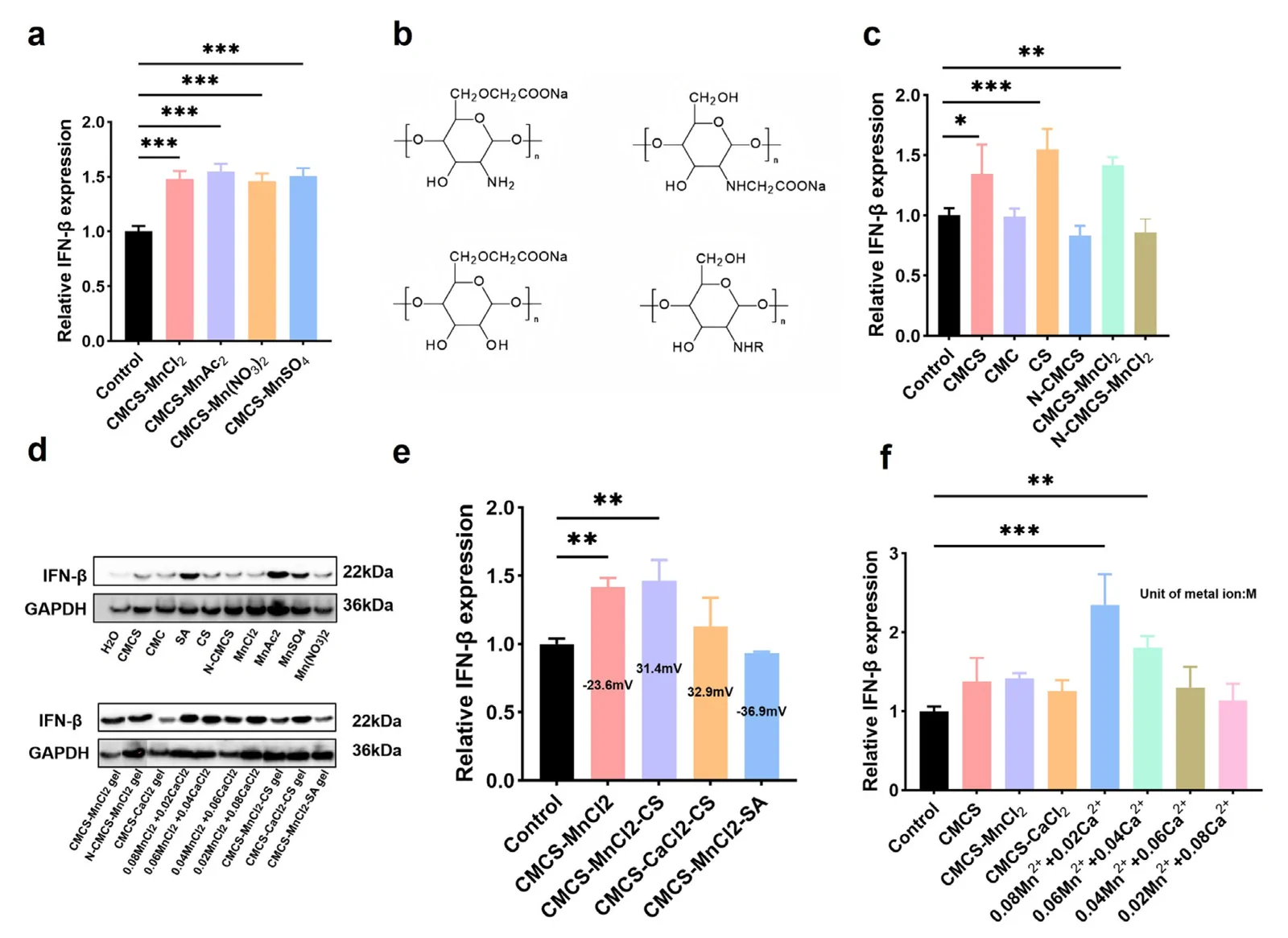
(**a**) Effects of gels prepared with different anions on IFN-β expression (*n* = 3). (**b**) Structural formulas of CMCS, CMC, CS, and N-CMCS. (**c**) Effects of CMCS, CMC, CS, N-CMCS, CMCS-MnCl_2_ gel and N-CMCS-MnCl_2_ gel on IFN-β expression (*n* = 3). (**d**) Western blot bands of IFN-β under corresponding treatment conditions. (**e**) The effect of gels with different surface potentials on IFN-β expression (*n* = 3). (**f**) Effect of Mn^2+^ content in the gel on IFN-β expression (*n* = 3). Data are presented as mean ± SD. *p*-values were calculated using ordinary one-way ANOVA with Dunnett’s multiple comparisons test. * *p* < 0.05, ** *p* < 0.01, *** *p* < 0.001.

To investigate the influence of anion type on the IFN-β induction of the gel, manganese salts containing four different anions: MnCl_2_, MnSO_4_, MnAc_2_, and Mn(NO_3_)_2_, were employed for gel fabrication. The IFN-β expression level in the CMCS-MnCl_2_ gel group and CMCS-MnSO_4_ gel group was approximately 1.5-fold that of the control group, while the CMCS-MnAc_2_ and CMCS-Mn(NO_3_)_2_ gel groups reached approximately 1.4-fold and 1.5-fold that of the control group, respectively (Figure 6a). No statistically significant difference was observed in the IFN-β-inducing capacity among gels prepared with different anions, and all values fell within the normal experimental variation range. The experimental results indicate that the anion present in the gel has minimal impact on its IFN-β induction; rather, the immunological performance of the gel is primarily determined by Mn^2+^ and CMCS.

Four polysaccharides with similar backbone structures (CMCS, CMC, CS and N-CMCS) were used to stimulate cells (Figure 6b). The IFN-β expression level in the CMCS group was approximately 1.4-fold that of the control group; in the CMC group, it was about 1.0-fold; in the CS group, about 1.6-fold; and in the N-CMCS group, approximately 0.8-fold (Figure 6c). The experimental results indicate that CS and CMCS with amino groups on their molecular chains significantly promote IFN-β expression, whereas CMC and N-CMCS, which lack free amino groups, exhibit no clear stimulatory effect. This suggests that free amino groups are crucial for the immunological activity of CMCS. When carboxymethyl substitution occurs at the N-position of chitosan, the amino structure is disrupted, and the resulting N-CMCS loses immunostimulatory activity. The IFN-β expression level in the N-CMCS-MnCl_2_ gel group was only 0.8-fold that of the control group. By contrast, the CMCS-MnCl_2_ gel group increased IFN-β expression to approximately 1.5-fold that of the control group, while the CMC-MnCl_2_ gel group showed no obvious enhancement.

To explore whether surface charge regulates the gel’s immune activity, hydrogels with different surface potentials were fabricated. The IFN-β expression in the CMCS-MnCl2 gel group was 1.4-fold that of the control group; in the CMCS-MnCl_2_-CS gel group, it was 1.5-fold; in the CMCS-CaCl_2_-CS gel group, it was 1.2-fold; and in the CMCS-MnCl_2_-SA gel group, it was 0.9-fold (Figure 6e). Both SA and CMCS shell layers exhibited negative potentials, while the CS shell layer exhibited a positive potential. Adding a CS shell layer increased hydrogel-induced IFN-β expression, whereas adding an SA shell layer reduced it, indicating that IFN-β induction depends on the outer shell layer. Hydrogels with CS or CMCS outer shell layers, both of which can trigger immune responses, showed greater IFN-β induction. However, shell-layer surface potential did not significantly affect IFN-β induction. Given the mechanism by which chitosan stimulates the cGAS-STING pathway, the hydrogel may act primarily after entering cells.

A series of mixed-ion hydrogels with varying Mn^2+^ concentrations were prepared using mixed CaCl_2_ and MnCl_2_ solutions to examine how Mn^2+^ content affects the immunostimulatory activity of the gel. The IFN-β induction of the CMCS-MnCl_2_ gel group was 1.3-fold that of the control group, the CMCS-CaCl_2_ gel group was 1.1-fold that of the control group, and the CMCS group was 1.2-fold that of the control group (Figure 6f). The effect of the CMCS-CaCl_2_ gel group was similar to that of the CMCS group, indicating that Ca^2+^ cannot directly induce cGAS–STING immune activation within this system. As the Mn^2+^ concentration decreased, the IFN-β induction declined correspondingly, reaching 2.2, 1.7, 1.3, and 1.1-fold that of the control group, respectively (Figure 6f). At higher Mn^2+^ contents, Ca^2+^ appeared to enhance Mn^2+^-induced IFN-β expression to some extent. As the Mn^2+^ content decreased, the hydrogel’s immunostimulatory activity gradually weakened, and the effect of the 0.02 mol/L MnCl2 + 0.08 mol/L CaCl_2_ gel group was similar to that of the CMCS-CaCl_2_ gel group, indicating that Mn^2+^ in the gel had little effect at this concentration. These results indicate that the main components contributing to the gel’s IFN-β induction are Mn^2+^ and CMCS, that Ca^2+^ does not act as an immune agonist in this system, and that a reduction in Mn^2+^ content leads to a decrease in the hydrogel’s immunostimulatory activity.

Ca^2+^ in the gel modulates the hydrogel’s immunostimulatory activity, which may arise from the effects of Ca^2+^ on gel microstructure. As demonstrated by the FT-IR characterization of single-ion gels described above, shifts in the characteristic carboxylate bands confirm that both Mn^2+^ and Ca^2+^ can crosslink CMCS molecular chains. However, compared with Mn^2+^, Ca^2+^ has a lower binding affinity for CMCS, resulting in more loosely packed microparticle structures [28]. Accordingly, we speculate that partial substitution of Mn^2+^ by Ca^2+^ may loosen the crosslinked network of mixed-ion hydrogels and thereby facilitate Mn^2+^ release from the gel.

Previous electrophysiological studies [29] have reported that Mn^2+^ can permeate voltage-gated calcium channels because Mn^2+^ and Ca^2+^ have similar ionic radii and charges. We therefore hypothesize that extracellular Mn^2+^ competes with Ca^2+^ for binding sites within the pores of these channels. Low concentrations of Ca^2+^ may promote cellular uptake of Mn^2+^ via this pathway. This hypothesis requires further experimental validation. The enhanced immune response observed at a low proportion of Ca^2+^ indicates that comparable immunostimulation can be achieved with reduced Mn^2+^ dosage. This finding suggests a way to reduce Mn^2+^ dosage and limit the risk of Mn^2+^ bioaccumulation.

HeLa cells were selected for cellular experiments in this study to assess proteins associated with the cGAS-STING pathway because this cell line retains an intact cGAS-STING pathway and yields high amounts of protein, facilitating analysis of the activation mechanism using Western blot (WB) phosphorylation assays. This model has been widely used to study Mn-triggered cGAS-STING activation [9,30,31].

Nevertheless, this study has several limitations. First, HeLa is a tumor-derived cell line rather than a professional innate immune cell type. Results from HeLa cells only confirm that the material can activate the cGAS-STING pathway but cannot independently establish immune activation. Second, IFN-β was used as the immune readout. While CMCS-Mn gel efficiently induces type I interferon responses and shows cGAS-STING activation potential, IFN-β can be induced by multiple innate immune and cellular stress pathways. Therefore, increased IFN-β alone cannot confirm specific activation of cGAS-STING signaling. Future studies will examine phosphorylation of upstream cGAS-STING pathway proteins and assess immune responses in macrophage cell lines (RAW264.7 and THP-1).

## 3. Materials and Methods

### 3.1. Materials

MnCl_2_·4H_2_O (analytical grade AR), CMCS (Aladdin, Cat. C304739, degree of substitution ≥ 90%), CMC (Aladdin, Cat. C104977-25g, degree of substitution = 0.9, MW 700000), SA (viscosity ≥ 0.02 Pa·s), CS (degree of deacetylation, 80.0–95.0%; viscosity, 0.05–0.80 Pa·s), HAc (content, 36–38%), EDTA-2Na, H_2_O_2_ (content ≥ 30%), Tris-HCl buffer, Tween 20 and NaCl were obtained from Sinopharm Chemical Reagent Co., Ltd. (Shanghai, China). HNO_3_ was purchased from Sigma Aldrich (St. Louis, MO, USA). An IFN-β polyclonal antibody was purchased from Ruixin Biotech Co., Ltd. (Quanzhou, China).

### 3.2. Cell Lines

HeLa cells obtained from the Microbiology and Immunology Laboratory of Huazhong Agricultural University (Wuhan, China) were cultured in RPMI-1640 medium containing 10% FBS and 1% penicillin/streptomycin at 37 °C in 5% CO_2_.

### 3.3. Determination of Mn^2+^ Content in Gels

After freeze-drying, the hydrogel samples were digested using a high-throughput microwave digestion system (JUPITER-B, Sineo Microwave Chemistry Technology Co., Ltd., Shanghai, China), and the corresponding Mn^2+^ content was determined using an atomic absorption spectrophotometer (WFX-200, Hitachi Scientific Instruments (Beijing) Co., Ltd., Beijing, China).

### 3.4. Preparation of CMCS-Mn Hydrogel

A 0.02 mol/L MnCl_2_ solution was added dropwise to a 1 mg/mL CMCS solution using a syringe under ultrasonication. For every 20 mL of CMCS solution, 1 mL of MnCl_2_ solution was added; these volumes can be scaled proportionally. After the MnCl_2_ solution had been completely added, ultrasonication was continued for 5 min at a power of 100 W.

### 3.5. Characterization of CMCS-Mn

FT-IR spectra of CMCS-Mn were measured using a Nicolet iS 50 spectrometer Nicolet iS50 spectrometer (Thermo Fisher, Waltham, MA, USA) (USA) over the range of 400 to 4000 cm^–1^ with 32 scans. A Hitachi SU 8010 scanning electron microscope (Hitachi, Tokyo, Japan) was used to characterize the surface morphology of CMCS-Mn. The mean diameter and size distribution of CMCS-Mn were measured by a Winner 802 particle size analyzer (China) Winner 802 particle size analyzer (Winner Instruments, Jinan, China). The specific surface area of CMCS-Mn was measured using an ASAP 2000 specific surface area and porosity analyzer (USA) ASAP 2000 analyzer (Micromeritics, Norcross, GA, USA). X-ray photoelectron spectroscopy (XPS) measurements were performed on an ESCALAB 250Xi XPS spectrometer (Thermo Fisher, Waltham, MA, USA) using Al Kα radiation (1486.6 eV).

### 3.6. Study of Mn^2+^ Release from CMCS-Mn

Mn^2+^ release behavior of CMCS-Mn hydrogel was investigated in DMEM-buffered medium at different pH values (pH 5.5, 6.8 and 7.4). A 1 g sample of CMCS-Mn hydrogel was dispersed in 30 mL of DMEM medium and incubated on a shaker at 37 °C and 100 rpm. Samples were collected at 0 h, 0.5 h, 1 h, 2 h, 4 h, 6 h, 8 h, 10 h, 12 h, and 14 h, with 2 mL collected each time. The amount of Mn^2+^ released during each time interval was determined using a WFX-200 atomic absorption spectrophotometer.

### 3.7. Effect of CMCS-Mn on Innate Immunity

CCK-8 assay was performed to verify the biosafety of CMCS, Mn^2+^ and their combinations within the tested concentration range. HeLa cells treated with different samples were incubated at 37 °C for 12 h at an Mn^2+^ stimulation concentration of 5 μg/mL. IFN-β expression was then assessed by Western blotting.

### 3.8. Statistical Analysis

All data were analyzed using GraphPad Prism 11.0 and presented as mean ± standard deviation (SD). The sample size for each analysis was given in the corresponding figure caption. Differences between two groups were evaluated using an unpaired *t*-test, while multiple groups were compared using ordinary one-way ANOVA with Dunnett’s multiple comparisons test. Differences were considered significant at * *p* < 0.05, ** *p* < 0.01, *** *p* < 0.001.

## 4. Conclusions

In this study, CMCS was used as the carrier to prepare a CMCS-Mn hydrogel through interactions between CMCS and Mn^2+^, thereby controlling Mn^2+^ release while using the immunological activity of both components. The hydrogel forms primarily through coordination bonds between the amino and carboxyl groups of CMCS and Mn^2+^. The results showed that CMCS and Mn^2+^ exhibit synergistic immunological effects, and that the CMCS-Mn hydrogel enhances this synergistic effect. The duration of the hydrogel’s immunological activity correlated with the sustained-release profile of Mn^2+^ within the gel. Anion type in hydrogels prepared with different manganese salts had little effect on IFN-β expression, indicating that Mn^2+^ plays a major role in the gel’s immunological function. Comparison of several structurally similar polysaccharides demonstrated that the amino group is the critical functional group responsible for CMCS’s immunological activity.

## Figures and Tables

**Table 1:** Effect of CMCS and Mn^2+^ concentrations on composition.

CMCS (mg/mL)	MnCl_2_ (mol/L)	Dry Weight of Gel (g)	Manganese Content (mg)	Mass Percentage of Mn (%)
1	0.02	0.153	1.488	0.969
	0.05	0.140	1.273	0.909
	0.1	0.145	1.413	0.974
	0.2	0.144	1.328	0.922
	0.5	0.165	1.696	1.027
0.5	0.1	0.077	0.765	0.993
	0.2	0.077	0.783	1.017

## Data Availability

The raw data generated in this study are available from the corresponding author upon reasonable request.

## Supplementary Materials

The following supporting information can be downloaded at: https://www.mdpi.com/article/10.3390/molecules31213891/s1, File S1: Raw SEM images; File S2: Original western blot images.
